# Applications of food science innovations in sports nutrition: from laboratory research to endurance sports practice

**DOI:** 10.3389/fnut.2026.1784740

**Published:** 2026-03-23

**Authors:** Meiying Ma, Guobao Yan, Guoyuan Huang

**Affiliations:** 1 Physical Education Department, Lanzhou University of Finance and Economics, Lanzhou, Gansu, China; 2 College of Physical Education, Sichuan Agricultural University, Ya’an, Sichuan, China; 3 Ya’an Key Laboratory of Sports Human Science and National Physical Fitness Promotion, Ya’an, Sichuan, China

**Keywords:** endurance sports, food science, natural ingredients, personalized nutrition, sports nutrition

## Abstract

Cutting-edge innovations in food science are driving sports nutrition from traditional supplementation toward a new paradigm based on molecular mechanisms and personalized interventions. This article illustrates the full chain of research advances in this field, from the laboratory to sports practice. It focuses on how microencapsulation, nanotechnology, and other processing technologies can overcome bottlenecks in the stability and delivery of active ingredients, thereby significantly improving their bioavailability. Furthermore, with the help of metabolomics and other omics techniques, it reveals the molecular pathways through which natural active ingredients regulate metabolism and alleviate exercise-induced fatigue and injury. In addition, the paper critically assesses the heterogeneity of current nutritional supplementation strategies in different sports and individual athletes, as well as the challenges faced by the industry in terms of scientific evidence and regulation. Looking to the future, the article constructs a new framework for precision sports nutrition that incorporates artificial intelligence, multi-omics-guided personalised nutrition, and sustainable manufacturing technologies, providing forward-looking insights to empower athletes to achieve peak performance and long-term health.

## 1 Introduction

In recent years, sports nutritional supplements have garnered significant attention for their roles in enhancing athletic performance, accelerating recovery, and reducing exercise-induced injuries, particularly among endurance athletes (1, 2). Research indicates that sports nutritional supplements help athletes achieve better performance in training and competition by providing additional energy sources, promoting muscle recovery, and enhancing endurance (3). For instance, *β*-alanine effectively improves high-intensity exercise performance, while creatine, widely used in short-duration, high-intensity activities, has demonstrated significant efficacy (4). The global sports nutrition market continues to expand, with research data indicating that the market size reached $33.6 billion in 2020, demonstrating steady growth (5). For instance, certain amino acids and compounds have been shown to enhance performance in high-intensity exercise, while supplements like creatine are widely used in strength training. The global sports nutrition market continues to expand, reflecting broad demand. However, controversy persists regarding the actual efficacy of many products compared to their labeled claims (6, 7). Therefore, scientific research on sports supplements is of paramount importance.

Continuous innovation in research methods has led to increasingly refined evaluation systems for sports nutrition products (8). The application of emerging technologies such as metabolomics and proteomics enables researchers to gain a deeper understanding of the mechanisms through which nutritional products affect human metabolic pathways (9). At the same time, the implementation of standardized double-blind controlled trials provides more reliable scientific evidence for assessing the efficacy of nutritional products (10). Advances in food science have brought new opportunities for the development of sports nutrition products (11). Targeted design of bioactive peptides, the application of microencapsulation technology, and the development of novel delivery systems have significantly improved the bioavailability and stability of nutritional products (12, 13). Studies have shown that natural ingredients (such as nitrates in beetroot extract) can significantly enhance endurance performance (14). In addition, the optimization of fermentation processes and innovations in functional food formulations have provided new ideas for the development of sports supplements (15). Safety assessment has become a critical component in the development of sports supplements (16). By establishing a comprehensive safety evaluation system that includes acute toxicity testing, long-term intake impact assessment, and studies on nutrient interactions, the safety of supplement use is ensured (17). During high-intensity training, controlling supplement dosage and timing is particularly crucial (18).

The rise of personalized nutrition is driving future sports nutrition supplements to focus on individual differences, providing customized nutritional plans based on athletes’ physiological characteristics and needs (19). The application of genetic testing technology allows researchers to develop more precise supplementation strategies according to individual metabolic characteristics (20). Such personalized approaches not only help enhance athletic performance but also effectively reduce the risk of sports injuries (21).

With the continuous advancement of food science, the field of sports nutrition supplements also demonstrates significant innovation potential. Emerging areas such as natural food ingredients, functional foods, and personalized nutrition are reshaping the research and application landscape of sports supplements (22). This article aims to the research progress in sports supplements, explore their roles in enhancing athletic performance, accelerating recovery, and reducing sports injuries, and analyze the innovative potential of food science in this field, with the hope of providing important references for research and practice in sports nutrition supplements.

## 2 Fundamental principles: endurance physiology and nutritional supplementation

### 2.1 Physiological demands of endurance exercise

#### 2.1.1 Energy metabolism and substrate utilization

**Figure 1:**
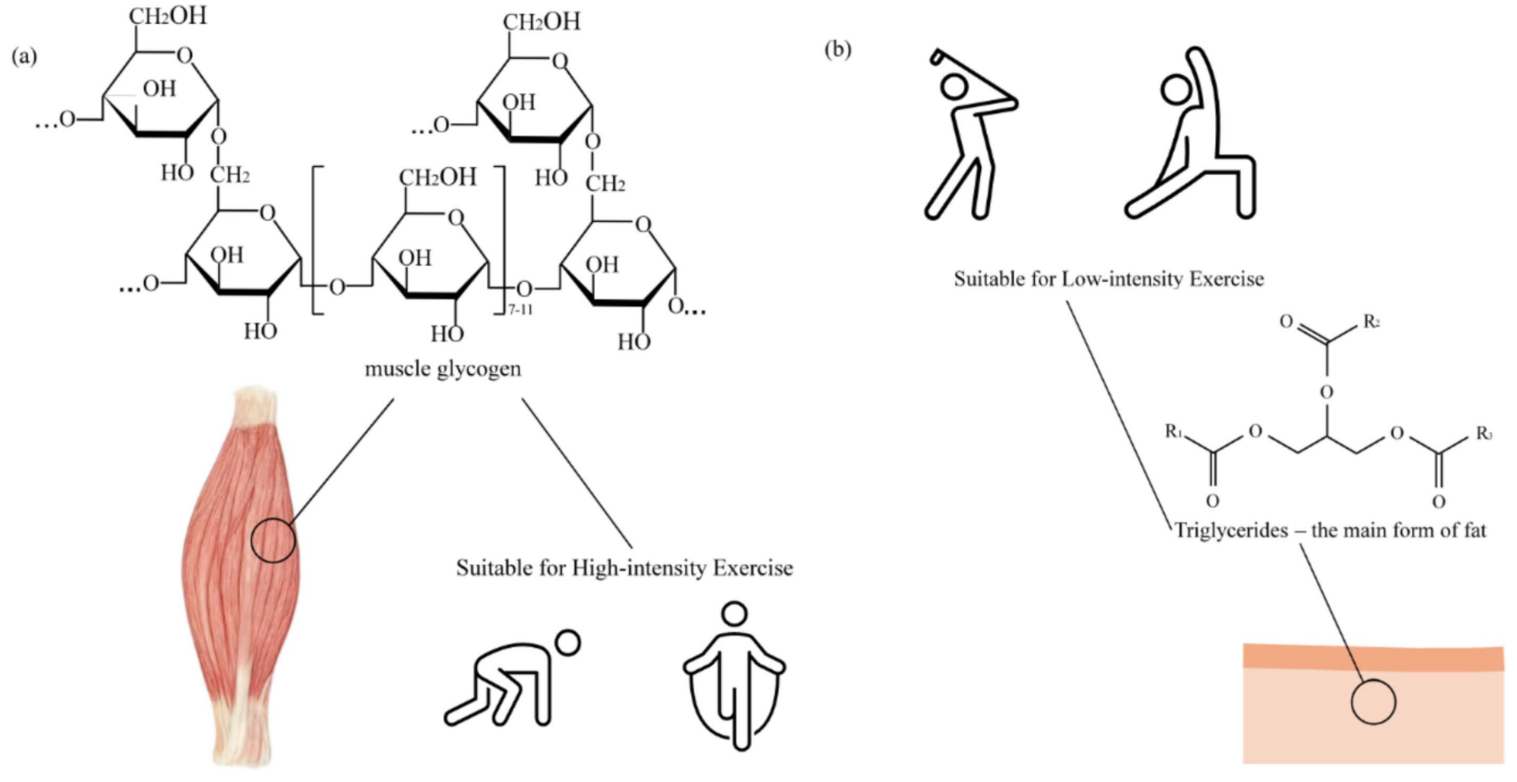
Schematic representation of primary energy substrate utilization during exercise. **(a)** Glycogen serves as the predominant fuel during high-intensity exercise. **(b)** Triglycerides (fats) become the major energy source during low-intensity, prolonged activities. The chemical structures are depicted for illustrative purposes.

Endurance exercise places extremely high demands on energy metabolism. During prolonged exercise, the body’s energy reserves are rapidly depleted. Therefore, maintaining a continuous energy supply is crucial for enhancing athletic performance and delaying fatigue. The primary energy sources for endurance exercise include carbohydrates, fats, and proteins. Among these, carbohydrates (such as muscle glycogen) and fats play different roles depending on exercise intensity and duration. As shown in Figure 1a, glycogen is rapidly depleted during high-intensity exercise; whereas, as shown in Figure 1b (23), fat becomes the main energy source during low-intensity endurance activities. To ensure athletes maintain high performance levels during prolonged exercise, although proteins and amino acids are important components of the diet, research indicates that additional supplementation does not directly enhance endurance or strength. In endurance exercise, moderate supplementation of carbohydrates and sports drinks helps maintain energy supply and delay the onset of fatigue (23).

#### 2.1.2 Lactate threshold and acid–base balance

The lactate threshold refers to the critical point during exercise when blood lactate concentration begins to rise significantly, serving as an important indicator of endurance performance. As exercise intensity increases, the accumulation of lactate leads to acidosis, thereby accelerating the onset of fatigue (24). Therefore, improving the lactate threshold is a key objective of endurance training. Studies have shown that supplementation with specific sports nutrition agents (particularly beta-alanine, amino acids, and carbohydrates) can effectively delay lactate accumulation and enhance the lactate threshold (25). These nutritional agents can improve muscle buffering capacity, helping athletes maintain lower lactate levels at higher intensities, thereby extending endurance performance and delaying the onset of fatigue (26).

#### 2.1.3 Oxygen utilization and cardiovascular efficiency

Efficient oxygen utilization is crucial for endurance performance, particularly during sustained low-to-moderate intensity exercise, where oxygen supply directly determines an athlete’s endurance level (27). Oxygen transport in the human body is primarily achieved through convection and diffusion, and its uptake and transport efficiency involve multiple factors, including cardiac output, the oxygen-carrying capacity of hemoglobin, and the efficiency of oxygen utilization by tissue cells. When the diffusion distance is long and the pressure gradient is low, the oxygen diffusion rate is slower; conversely, when the diffusion distance is short and the pressure gradient is high, the oxygen diffusion rate is faster. This diffusion characteristic is essential for understanding the regulatory mechanisms of tissue oxygen supply (28).

**Figure 2:**
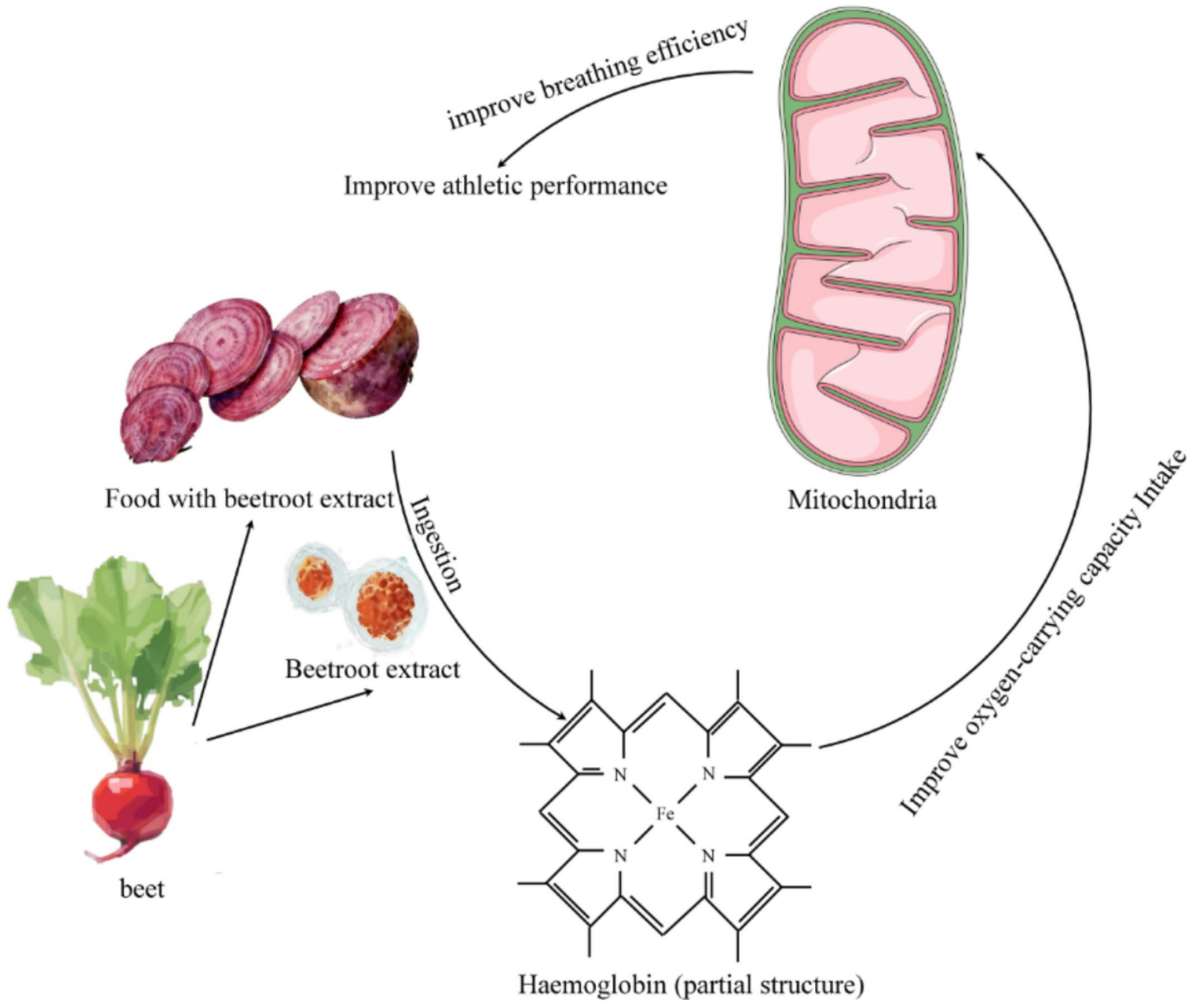
Proposed mechanism of action for dietary nitrate (e.g., from beetroot extract) on exercise performance. The diagram illustrates the nitrate-nitrite-nitric oxide (NO) pathway, leading to enhanced blood flow, improved mitochondrial efficiency, and reduced oxygen cost of exercise.

Research indicates that supplementing with nutrients such as nitrates can enhance exercise performance. For example, as shown in Figure 2, nitrate-rich beetroot extract can improve oxygen utilization efficiency. Supplementing with iron and vitamins helps enhance the oxygen-carrying capacity of hemoglobin. Ingested nitrates are converted through the nitrate-nitrite-nitric oxide metabolic pathway, increasing the bioavailability of nitric oxide, which in turn reduces ATP consumption during muscle contraction, improves mitochondrial respiratory efficiency, increases muscle blood flow, and ultimately enhances exercise performance.

### 2.2 Definition, classification, and usage patterns of sports supplements

Sports nutritional supplements are typically defined as foods or food components used to enhance athletic performance, promote recovery, or meet nutritional needs (29). These products can be classified based on their chemical characteristics (such as proteins, carbohydrates, vitamins, minerals, etc.) and their functions (30).

As shown in Table 1, sports supplements can be categorized according to their impact on athletic performance, including: endurance-enhancing types (such as *ω*-3 fatty acids and *β*-carotene), muscle maintenance and recovery types (such as EPA ethyl ester), situation-specific enhancement types (such as vitamin E), body composition regulation types (such as Garcinia cambogia extract), and functional optimization types (such as vitamin D). These supplements can also be further classified based on chemical properties (such as proteins, carbohydrates, vitamins, minerals, etc.) and functional characteristics (such as energy enhancers, buffers, anti-inflammatory agents) (31).

**Table 1:** Common sports supplements: dosage, duration, exercise effect and safety.

Supplement name	Daily dose	Supplementation duration	Exercise effect	Safety considerations
Omega-3 fatty acid	0.84–1 g	5.3–7.4 years	Improves endurance but has no muscle building effect (159)	May increase the risk of bleeding
Omega-3 Ethyl Esters	4 g	4.9 years	Helps maintain muscles after exercise injury (160)	Requires monitoring by a doctor
Vitamin E	400–600 IU	7–10 years	May be beneficial in altitude training and short-term performance (161)	May increase the risk of heart failure
Beta-carotene	20–30 mg	4–6 years	Positively affects endurance running performance (162)	Not recommended for smokers
Chromium	400–1,000 μg	12–17 weeks	No significant effect on body composition and muscle mass (163)	May cause nausea and dizziness
Chitosan	1–2.5 g	12–17 weeks	Combined with weight training can improve aerobic capacity and inflammation indicators (164)	Generally safe
Garcinia cambogia	1–2.8 g	8–12 weeks	Mainly affects body weight by regulating fat metabolism and satiety (165)	May be toxic to the liver
Vitamin D	400–800 IU	varies	May improve exercise performance by improving muscle function and testosterone levels (166)	Generally safe at recommended doses

Sports supplements are widely used among athletes, with elite athletes showing significantly higher usage rates than non-elite athletes, ranging from 40% to 100% (32). Among young athletes, the usage rate is as high as 82.2% (33). Notably, most users take multiple supplements simultaneously. For example, 82.6% of high-level athletes in the UK use more than two supplements concurrently (34). However, supplement use may pose health risks and issues related to doping tests (35). Athletes’ beliefs and attitudes also influence their supplement use behavior (36). Research indicates that athletes’ decisions to use supplements are influenced by multiple factors, including personal attitudes, subjective norms, and perceived behavioral control (37). Male athletes are more inclined than female athletes to adopt performance-enhancing methods (38). It is noteworthy that strong beliefs in the effectiveness of supplements may increase athletes’ tendency to use doping substances (39). Studies have found that although up to 87.5% of athletes use nutritional supplements, most users are unaware of their active ingredients, side effects, and mechanisms of action (40). This knowledge gap underscores the importance of enhancing supplement education.

Certain sports supplements are believed to significantly enhance athletic performance. Among the most popular, creatine supplements improve high-intensity exercise performance by increasing intracellular creatine concentration, while also potentially promoting recovery and preventing injuries, with their long-term safety having been confirmed (41). Similarly, systematic studies have demonstrated that whey protein supplements significantly improve athletes’ respiratory exchange ratio, average power, and other metrics (42). Different types of supplements may have specific functions: for instance, arginine can improve cardiovascular function and maximal oxygen uptake, branched-chain amino acids can enhance reaction speed, and vitamin D helps regulate hormone levels related to injuries (43).

However, many commercially available pre-workout supplements often contain “proprietary blends” of multiple ingredients, with limited or conflicting evidence for the effectiveness of most components. These products may also cause adverse reactions such as gastrointestinal discomfort and arrhythmia (44). Although sports nutrition products play only a supplementary role in overall nutrition plans, their usage is prevalent across all levels of sports, with coverage rates as high as 40% to 100%. Despite the widespread advocacy of the “food first” principle, supplement use remains indispensable in specific situations such as tight training schedules, limited food storage conditions, and digestive comfort (45).

### 2.3 Differences between functional foods and traditional dietary supplements

The primary distinction between functional foods and traditional dietary supplements lies in their comprehensive nutritional composition and health benefits, as shown in Table 2. Functional foods not only provide essential nutrients such as proteins, fats, carbohydrates, vitamins, and minerals but also offer additional physiological functions or health benefits. These benefits typically include enhancing immune function, promoting cardiovascular health, regulating metabolism, boosting antioxidant capacity, and even reducing the risk of certain diseases (46). For example, antioxidant-rich blueberries, anti-inflammatory turmeric, and deep-sea fish oil rich in *ω*-3 fatty acids are all typical functional foods (47). While meeting basic nutritional needs, they can also improve athletes’ health and competitive performance (48, 49).

**Table 2:** Comparison of characteristics between functional foods and traditional dietary supplements.

Feature comparison items	Functional food	Traditional supplements
Nutritional characteristics	Comprehensive nutrient composition (including essential nutrients and bioactive substances)	Single specific nutrients (e.g., vitamins, minerals, amino acids)
Main purpose	Provides comprehensive nutritional support and additional health benefits	Compensate for specific nutrient deficiencies or enhance specific functions
Mechanism of action	Multiple mechanisms of action (e.g. anti-oxidant, anti-inflammatory, metabolic regulation, etc.)	Targeted mechanism of action
Health benefits	Long-term health promotion (improves immune function, cardiovascular health, metabolic regulation)	Short-term goal improvement (improved athletic performance, muscle building, accelerated recovery)
Safety considerations	Natural ingredients, strictly regulated, low risk of side effects	Risk of overdose, systemic burden, long-term dependency possible
Representative examples	Blueberries (anti-oxidant), turmeric (anti-inflammatory), deep-sea fish oil (omega-3)	Vitamins, minerals, amino acids, proteins

In contrast, traditional supplements typically focus on specific nutrients, with the primary goal of supplementing single nutritional components such as vitamins, minerals, amino acids, or proteins to address deficiencies in athletes’ daily diets or enhance performance through specific ingredients (50). The effects of traditional supplements are often targeted at short-term goals, such as improving exercise performance, increasing muscle mass, or accelerating recovery. Although traditional supplements can provide rapid and significant results for athletes, long-term reliance on single supplements may pose potential health risks, such as excessive intake of specific components or placing a burden on other bodily systems (51).

With the development of sports nutrition, the potential of functional foods in the athletic field has gradually gained attention. Research indicates that functional foods not only meet athletes’ basic nutritional needs but also promote health and enhance performance through multiple mechanisms (52). For example, natural ingredients in specific functional foods can enhance the body’s antioxidant capacity, reduce exercise-induced inflammation, or optimize energy metabolism, thereby supporting athletes’ long-term health and competitive condition (53). The advantage of functional foods lies in their diverse nutritional components and lower risk of side effects, providing athletes with more comprehensive and balanced nutritional support. Compared to traditional supplements, functional foods offer significant safety advantages—their natural ingredients and standardized production processes are strictly regulated, ensuring a superior safety profile for users (54, 55).

## 3 Laboratory development of sports nutrition supplements

### 3.1 Advances in food processing technology

#### 3.1.1 Microencapsulation technology

**Figure 3:**
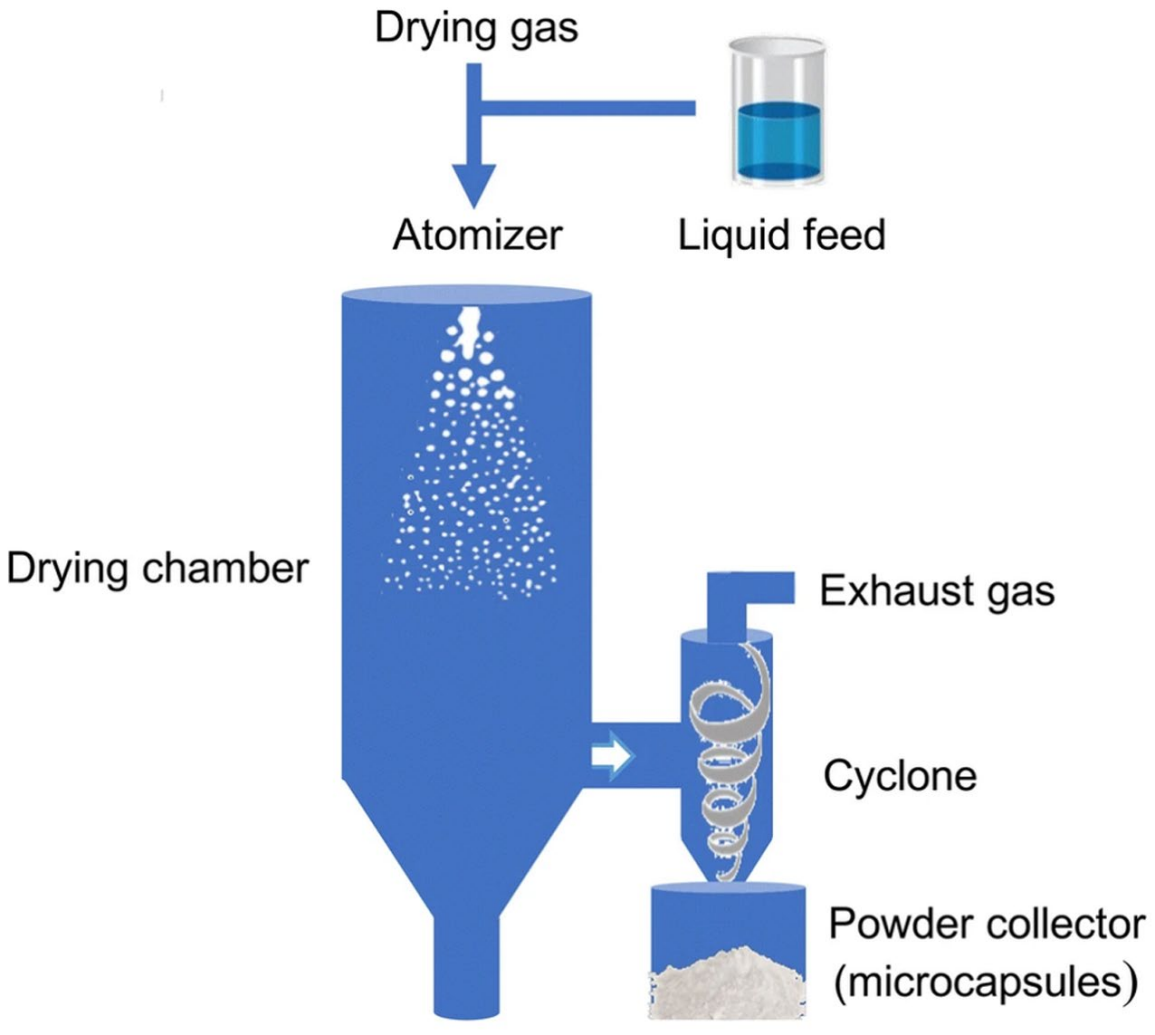
Schematic of the spray-drying microencapsulation process. The diagram outlines key steps: Formation of an emulsion or dispersion containing the active core material and wall polymer, atomization into droplets, and rapid solvent evaporation to form solid microspheres, thereby protecting the encapsulated ingredient. Adapted from Arenas-Jal et al. (56).

Microencapsulation technology plays a pivotal role in sports supplements by encapsulating active ingredients within polymer or lipid membranes, significantly enhancing their stability, bioavailability, and controlled release (56). Spray drying is the oldest and most widely used process for microencapsulation in the food industry. As shown in Figure 3, in this process, the mixture of the shell layer and the core material is dissolved, dispersed or emulsified in a solvent, and then atomized to form an aerosol. Subsequently, the solvent is removed through heating to form tiny solidified droplets (microspheres), thereby encapsulating the target compound (56, 57). This technology protects active ingredients from degradation in the gastrointestinal environment and improves their absorption efficiency in the small intestine (57). With advancements in multi-layered microparticle technology, the level of protection for active ingredients has been substantially enhanced, enabling them to overcome physiological barriers more effectively (58). For instance, microencapsulated vitamins and minerals exhibit improved intestinal absorption, thereby increasing the nutritional value and efficacy of sports supplements (59).

Furthermore, microencapsulation technology extends the shelf life of active ingredients by addressing issues related to oxidation or degradation, ensuring product stability during storage and transportation (60, 61). Additionally, this technology enhances the sensory properties of supplements by masking unpleasant flavors within the microcapsules, thereby improving consumer experience (62).

Microencapsulation technology not only enhances the functionality and stability of sports supplements but also meets consumer demands for high efficiency, convenience, and superior taste. It has become an indispensable technological innovation in the field of sports nutrition. However, the current efficacy assessment of microencapsulation technology in sports nutrition applications is mainly based on *in vitro* mechanistic studies and limited bioavailability human trials, and there is a lack of large-scale, long-term validation of efficacy in elite athlete populations.

#### 3.1.2 Nanotechnology enhances absorption and targeted delivery

**Figure 4:**
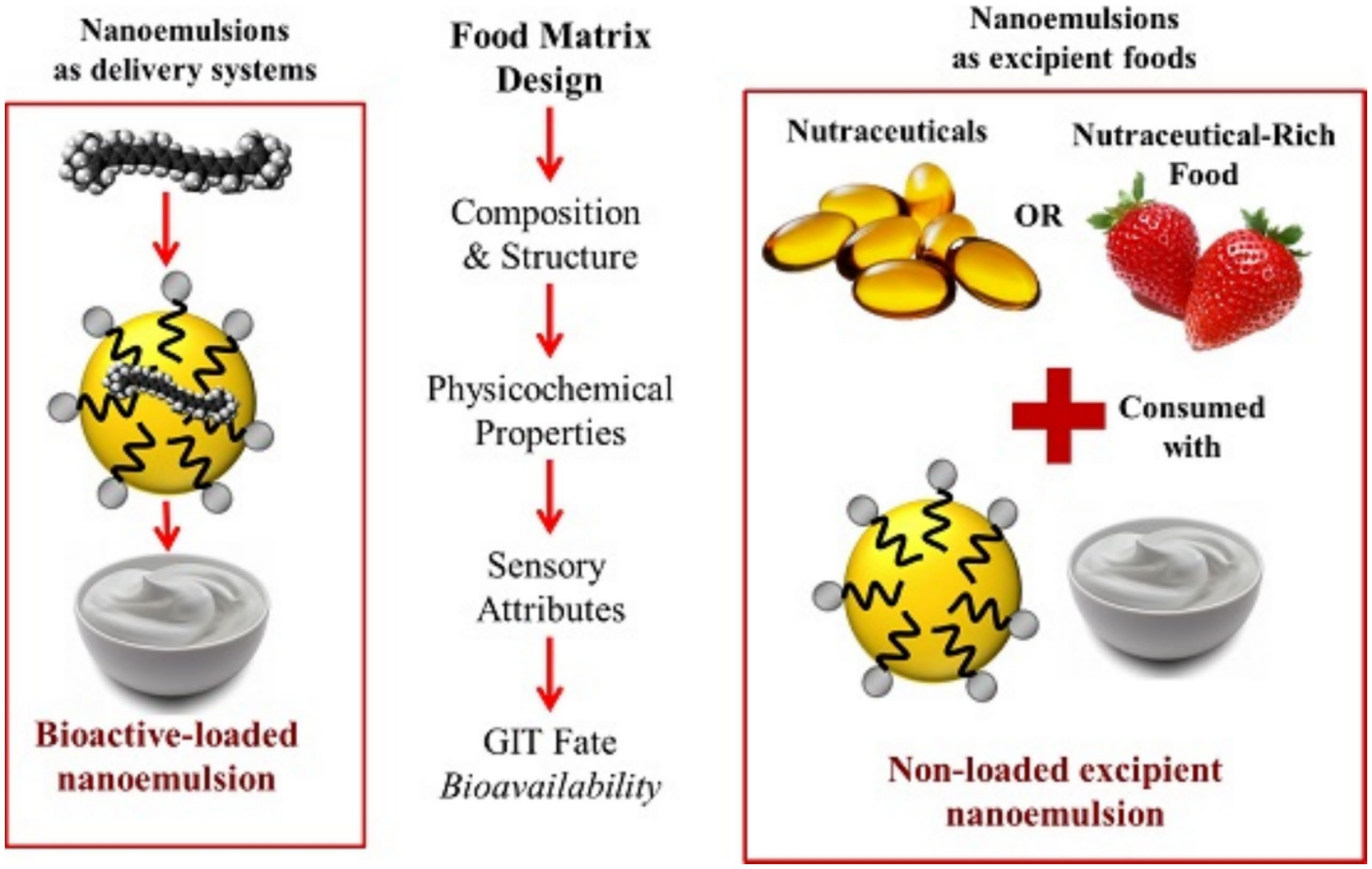
General scheme for preparing nanoemulsion-based delivery systems for bioactive compounds. Bioactive agents are dispersed within an excipient food formulation using high-energy methods (e.g., high-pressure homogenization, ultrasonication) to create kinetically stable droplets at the nanoscale, aiming to enhance solubility and bioavailability. Adapted from Salvia-Trujillo et al. (158).

Nanotechnology has introduced innovative opportunities for the advancement of sports supplements. By nano-sizing active ingredients, the surface area of these components is significantly increased, leading to a substantial improvement in their solubility and bioavailability (63, 64). In laboratory, the nanoemulsion food can be made by dispersing the bioactive agents into the excipient food formulation or co-ingested with an excipient food formulation (Figure 4). This implies that nano-sized nutrients can be absorbed more rapidly and efficiently by the body, enhancing the nutritional absorption efficiency and efficacy of supplements for athletes.

Additionally, nanotechnology can enhance the stability of sports supplements by preventing the oxidation or degradation of active ingredients during storage and use, thereby extending the product’s shelf life (65). Nanotechnology also demonstrates potential for targeted delivery, enabling the precise transport of active ingredients to specific tissues or organs. For example, anti-inflammatory or antioxidant components can be delivered directly to muscle tissues or joints. This targeted delivery capability may open new avenues for improving the effectiveness of sports supplements (66).

In summary, nanotechnology not only enhances the bioavailability and stability of active ingredients in sports supplements but also offers the potential for targeted delivery, paving the way for innovative approaches to optimizing athletic performance and recovery. It should be noted that research on the application of nanotechnology in sports nutrition is currently focused on preclinical modelling and proof-of-concept phases, and its targeted delivery efficacy and long-term safety in athletes have yet to be confirmed in human trials.

#### 3.1.3 Critical considerations, safety, and translational hurdles of nanotechnology

While the preceding section outlines the potential benefits of nanotechnology, a balanced and rigorous scientific review necessitates an equally critical examination of its limitations, safety concerns, and translational challenges—aspects currently underemphasized in the sports nutrition literature (67).

Toxicological Concerns and Unintended Biological Interactions: The very properties that enhance the functionality of nanoparticles—such as their high surface area-to-volume ratio and increased reactivity—also raise significant toxicological questions (68). Nanoparticles may induce oxidative stress, trigger inflammatory responses, or cause cellular damage in ways that bulk materials do not. These risks could be exacerbated in athletes undergoing intense physiological stress. Furthermore, the long-term fate of engineered nanomaterials within the human body, especially under chronic supplementation scenarios, remains largely unknown (69).

Regulatory Uncertainty and Characterization Challenges: The global regulatory framework for nanomaterials in food and dietary supplements is fragmented and lags behind technological innovation (70). Many jurisdictions lack specific pre-market safety assessment protocols for nano-enabled supplements, creating a “regulatory grey area” (71). This is compounded by significant technical hurdles in reliably characterizing nanoparticles within complex product matrices, including their size distribution, stability, and potential for aggregation, which complicates quality control and independent verification of safety claims (72).

Risks of long-term bioaccumulation: a critical and understudied risk is the potential for bioaccumulation (73). Due to their small size, nanoparticles may bypass normal metabolic and excretory pathways, accumulating in specific organs such as the liver, spleen, or kidneys over time (74). The chronic impact of such accumulation, particularly when combined with the physical demands of elite sport, represents a serious knowledge gap that must be addressed before widespread adoption can be recommended.

Lack of human clinical trials and the translational gap: as noted earlier, much of the supportive evidence is preclinical. There is a profound scarcity of robust, long-term human clinical trials, especially those conducted in athlete populations, to confirm efficacy and safety (75). The leap from promising laboratory results to proven, commercially viable, and safe products for athletes involves overcoming major hurdles in scalable manufacturing, cost-effectiveness, and demonstrating a clear risk–benefit advantage over conventional delivery systems.

In conclusion, the application of nanotechnology in sports nutrition is a double-edged sword. Its promising potential for enhancing nutrient delivery is tempered by substantial and unresolved questions regarding safety, regulation, and practical implementation. Future research must pivot towards targeted human studies with comprehensive safety monitoring, while industry and regulators collaborate to establish clear safety standards. A precautionary approach is advisable until these critical issues are adequately resolved.

#### 3.1.4 Fermentation technology enables the generation of bioactive components

Fermentation, technology demonstrates unique advantages in the development of sports supplements, serving as an effective approach to enhance product functionality and consumer acceptance. Through the fermentation process, not only can the nutritional value and bioavailability of raw materials be improved, but a variety of beneficial metabolites, such as amino acids, vitamins, minerals, and probiotics, can also be generated. These components provide significant benefits for athletes’ health and performance (76). For example, fermented soybean products are not only rich in high-quality protein but also undergo the degradation of anti-nutritional factors (such as phytic acid and protease inhibitors) during fermentation, significantly enhancing their digestibility and absorption. This enables athletes to more efficiently absorb essential nutrients (77).

Additionally, fermentation technology, through the metabolic activities of bacteria or yeast, can produce beneficial functional components such as B vitamins and short-chain fatty acids (78, 79). These components play a positive role in enhancing energy metabolism, promoting immune function, and reducing inflammatory responses (80). Therefore, sports supplements utilizing fermentation technology can not only improve the body’s nutritional status but also enhance post-exercise recovery and overall health.

### 3.2 The mechanism of action of new bioactive ingredients

**Figure 5:**
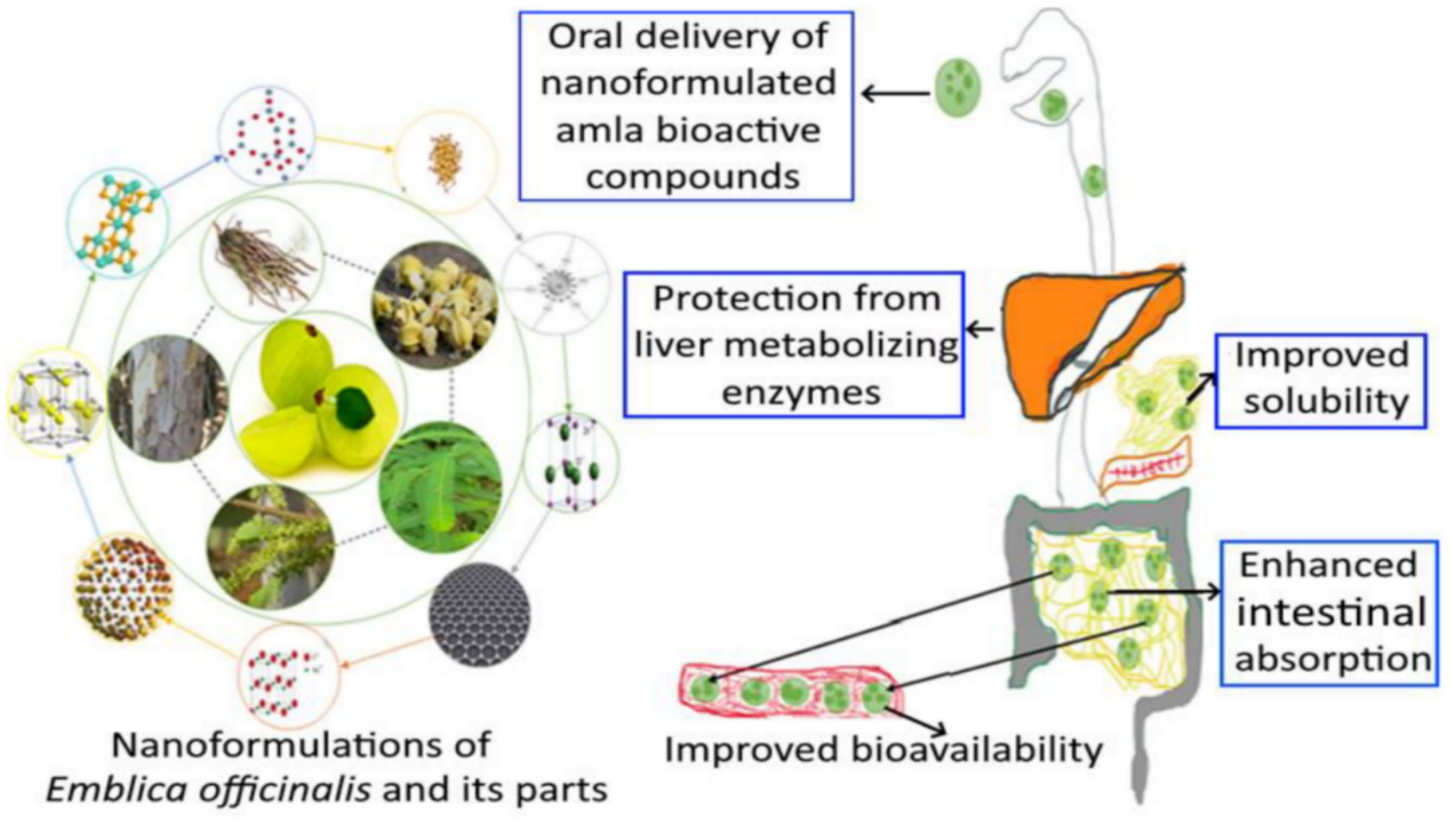
Conceptual diagram of a nanoparticle delivery system for gallic acid (from amla) and its proposed physiological journey. The figure depicts the design of a composite nanoparticle and its hypothesized pathway through the gastrointestinal tract, with an emphasis on targeted release in the colon. Adapted from Rachitha et al. (86).

The development of new sports nutrition supplements cannot be separated from a deep understanding of the mechanism of action of bioactive ingredients. Bioactive ingredients refer to compounds that occur naturally in food or are added through processing and have beneficial effects on human health (81). For example, polyphenols is widely present in fruits and vegetables, they offer multiple health benefits such as antioxidant, anti-inflammatory, and cardiovascular disease prevention properties (82–84). For instance, polyphenols can protect the liver through their antioxidant properties conferred by hydroxyl groups and may improve alcohol-induced liver disease by regulating the gut microbiota (85). For proteins and peptides, they play a significant role in regulating skeletal muscle function and can address issues such as muscle atrophy and sarcopenia associated with aging (86). To overcome these limitations, advanced delivery systems such as the microspheres and nanoparticles described above can be employed to enhance the bioavailability and targeted efficacy of these bioactive ingredients. For example, to enhance the water solubility of Amla and thereby increase its biological activity, gallic acid was extracted and isolated. Using a probe-type ultrasonic instrument and high-pressure homogenization, gallic acid nanoparticles were prepared by mixing monoglyceride of oleic acid (GMO), chitosan, and poloxamer 407. The research results indicated that nanoparticles could be designed and fabricated to facilitate the extraction, production, and sustained release of gallic acid, especially in the colon region (87) (see Figure 5).

#### 3.2.1 Efficacy and mechanisms of action of plant extracts

The application of plant extracts in sports nutrition is gaining increasing attention due to their rich content of various bioactive compounds, which can effectively enhance athletic performance and recovery. High-intensity exercise may lead to muscle damage, causing muscle weakness, pain, and reduced capacity for subsequent training and competition. Proper nutritional supplementation can accelerate the recovery process. Studies have shown that plant extracts such as green tea extract, curcumin, and ginseng possess multiple benefits, including antioxidant, anti-inflammatory, and energy metabolism-improving properties. These compounds modulate the body’s redox status, alleviating exercise-induced muscle damage and fatigue, thereby enhancing athletic performance. For example, curcumin reduces post-exercise muscle soreness and inflammation by inhibiting the release of inflammatory factors (such as IL-1β, IL-6, TNF-*α*), thus promoting muscle recovery (87). The polyphenols in green tea extract exhibit strong antioxidant effects, mitigating the negative impact of oxidative stress on athletes and significantly improving post-exercise recovery and muscle function (88). Ginseng, with its adaptogenic properties, excels in enhancing strength and endurance, boosting immunity, and counteracting excessive physical stress during exercise.

As shown in Table 3, different plant extracts have varying effects on exercise performance. Green tea extract (571 mg daily for 28 days) promotes fat oxidation, reduces body fat, and enhances exercise performance; curcumin (1.5 g daily for 28 days) primarily aids in post-exercise recovery, alleviating exercise-induced damage and soreness. Ginseng (1,125 mg daily for 35 days) mainly functions by modulating immune responses during exercise, but its impact on improving exercise performance is limited. These plant extracts demonstrate their respective application value in the field of sports nutritional supplements through different mechanisms of action.

**Table 3:** Effects of plant extracts on exercise performance.

Plant extract	Dosage	Duration of effect	Exercise effect
Green tea extract	571 mg/day	for 28 days	Increases fat oxidation; reduces body fat; improves athletic performance (87)
Curcumin	1.5 g/day	for 28 days	Reduces muscle damage after exercise; reduces post-exercise muscle soreness (88)
Ginseng	1,125 mg/day	for 35 days	Regulates the immune response to exercise; has a limited effect on exercise performance (167)

Additionally, plant extracts can improve fat metabolism, promote fat oxidation, and increase energy expenditure, helping athletes maintain a good energy balance during high-intensity training. This results in more sustained exercise performance. The multiple mechanisms of action of these plant extracts provide new directions for the development of sports nutrition products, particularly showing great application potential in enhancing exercise performance, accelerating recovery, and reducing exercise-induced injuries. The efficacy data for the botanical extracts listed in the table below are mainly derived from small-sample, short-term human trials or animal model studies, and their conclusions have yet to be consolidated in larger, more rigorous clinical trials with athletes.

#### 3.2.2 Relationship between probiotics and gut health

The application of probiotics in sports nutrition primarily involves improving gut microbiota composition, promoting intestinal health, and optimizing nutrient absorption. Gut health is crucial for athletes’ overall performance, as the gut is not only the core of the digestive system but also closely related to immune function and metabolism. Studies indicate that probiotics can enhance gut barrier function and reduce intestinal inflammation, thereby boosting athletes’ immunity and lowering infection risk (89, 90). They also improve digestive function and alleviate gastrointestinal discomfort such as diarrhea and stomach pain induced by exercise (91), which is particularly important for high-intensity training and endurance competitions.

Furthermore, probiotics enhance exercise performance by synthesizing metabolites such as short-chain fatty acids (SCFAs), which promote energy metabolism. These metabolites (acetate, propionate, butyrate) improve energy utilization efficiency, enhance gut barrier function, and increase propionate bioavailability, ultimately contributing to improved athletic performance (92). Short-chain fatty acids not only support fat oxidation and glucose metabolism but also improve intestinal energy balance, providing a sustained energy source for athletes.

Research has also found that certain probiotic strains can accelerate post-exercise recovery, reduce muscle damage caused by high-intensity exercise, alleviate exercise-induced fatigue, and promote muscle repair and regeneration (93). By modulating gut microbiota composition, probiotics can mitigate exercise-induced inflammatory responses and regulate physiological processes such as immune response, antioxidant activity, and protein synthesis, helping athletes quickly return to training condition.

### 3.3 Bioavailability and safety assessment

In the research and development of sports nutritional supplements, bioavailability and safety assessment are key steps to ensure product efficacy and consumer health (94). Bioavailability refers to the absorption, distribution, metabolism, and excretion of ingested nutrients in the body, directly determining the effectiveness of the supplement. Many natural ingredients (such as specific plant extracts, vitamins, and minerals) exhibit low bioavailability due to factors like food composition, digestive environment, or formulation design (95, 96). Therefore, innovative methods—including advanced formulation design, nanotechnology, and microencapsulation—are employed during the development process to enhance the absorption rate of these ingredients, thereby improving efficacy and maximizing the nutritional value of the supplements (97).

At the same time, safety assessment of sports supplements is equally important. To ensure product safety at recommended doses, rigorous clinical trials and long-term safety monitoring must be conducted (98). This not only helps identify potential side effects but also assesses the risk of adverse reactions under conditions of long-term use or high-dose intake. Through comprehensive scientific experiments, toxicological studies, and human clinical trials, the suitability and safety of supplements for different populations can be systematically evaluated, ensuring that the products do not negatively impact health.

## 4 Validation of the biological mechanisms of food ingredients in sports nutritional supplements

### 4.1 The role of metabolomics and biomarkers in mechanism elucidation

Metabolomics, as an emerging biological research tool, enables comprehensive analysis of metabolites and their changes within organisms, thereby revealing biological mechanisms under physiological and pathological conditions (99). In the study of sports nutritional supplements, metabolomics provides a powerful tool for understanding the biological effects of food components. By comparing the metabolic profiles of athletes before and after supplement use, researchers can identify biomarkers associated with athletic performance. These biomarkers reflect the impact of supplements on athletes’ metabolic processes, thereby assessing their effectiveness (100). For example, metabolites of certain amino acids and fatty acids (such as branched-chain amino acids [BCAAs] and *ω*-3 fatty acids) have been shown to be closely related to exercise endurance, muscle synthesis, recovery capacity, and fatigue resistance (101).

Metabolomics not only helps identify biomarkers related to athletic performance but also elucidates how sports supplements regulate biological processes such as energy metabolism, inflammatory responses, and oxidative stress (102). By integrating metabolomics with other “omics” technologies such as genomics and proteomics, researchers can gain a more comprehensive understanding of the mechanisms of action of food components in sports supplements and their potential biomarkers (103). This multidimensional research approach reveals the specific effects of different supplements at the cellular and molecular levels, providing a basis for developing personalized sports nutrition intervention programs. For example, customized supplement regimens can be designed for different types of athletes or exercise needs (such as endurance versus strength athletes) based on metabolic changes, thereby optimizing athletic performance and promoting rapid recovery.

### 4.2 Molecular mechanisms and metabolic responses of key natural ingredients

Beyond the aforementioned molecular mechanisms, an emerging research focus centers on plant-derived extracellular vesicles—membrane-bound nanovesicles naturally secreted by edible plants. These vesicles themselves are rich in bioactive substances such as nucleic acids, lipids, and proteins, possessing dual properties as both natural carriers and active ingredients (104). Certain plant-derived vesicles, such as those from ginger and grapes, possess inherent antioxidant and anti-inflammatory properties that directly regulate intracellular redox balance and inflammatory pathways (105). More importantly, they function as “smart” carriers, encapsulating and protecting digestion-sensitive functional molecules—such as specific polyphenols or microRNAs—to achieve precise delivery to target tissues or the gut microbiota. This presents an exceptionally compelling new paradigm for sports nutrition: leveraging natural nanocarriers derived from food matrices to synergistically enhance the stability, bioavailability, and overall efficacy of active ingredients in mitigating exercise-induced oxidative stress and inflammation. This approach represents an upgrade from “ingredient supplementation” to “systemic regulation”.

#### 4.2.1 Molecular mechanisms of natural ingredients and their metabolic responses in athletes

The following description of the molecular mechanisms of natural ingredients is based mainly on cellular and animal experiments and some small-scale human metabolism studies. More translational studies are needed to confirm the exact role of these mechanisms in complex human systems and in different types of exercise. The application of natural ingredients in sports nutritional supplements has become a research focus, particularly concerning their roles in enhancing athletic performance, promoting recovery, reducing exercise-induced injuries, and improving overall health. Natural compounds such as beetroot extract, curcumin, and Schisandra chinensis extract demonstrate significant physiological benefits by modulating athletes’ metabolic responses. As shown in Table 4, the nitrate in beetroot is converted into nitric oxide, promoting vasodilation and enhancing the delivery of oxygen and nutrients, thereby improving endurance (106). Curcumin, known for its antioxidant and anti-inflammatory properties, can mitigate exercise-induced oxidative damage and inflammatory responses, facilitating muscle repair and recovery (107). Schisandra chinensis extract enhances exercise endurance and anti-fatigue capacity by improving mitochondrial function and energy metabolism (108). Additionally, natural ingredients like goji berries also possess antioxidant and immune-boosting effects. Studies indicate that curcumin exerts its effects by inhibiting inflammatory factors; Schisandra chinensis improves energy metabolism by regulating the AMPK/PGC-1α signaling pathway; and goji berries provide multi-dimensional protective effects through their unique bioactive components, including LBP polysaccharides with molecular weights of 10–2,300 kDa and flavonoids at concentrations of 1.0–1.3 mg/g, offering comprehensive support for athletes’ training and recovery (109).

**Table 4:** Usage of natural ingredients in sports supplements.

Natural ingredients	Main active substances	Mechanism of action
Beetroot extract	Nitrates	Converted to nitric oxide, promotes vasodilation
Curcumin	Curcuminoids	Regulates the NF-κB pathway and inhibits inflammatory factors
Schisandra extract	Schisandrin	Regulates the AMPK/PGC-1α and SIRT1 pathways
Goji berry	LBPs Polysaccharides (10–2,300 kDa), flavonoids (1.0–1.3 mg/g)	Multiple protective effects

The molecular mechanisms of these natural ingredients involve multiple biological pathways: for example, curcumin alleviates inflammation by modulating the NF-κB pathway (110); beetroot enhances vasodilation and oxygen delivery via nitric oxide; and Schisandra chinensis improves mitochondrial function through the AMPK and SIRT1 pathways (111). These ingredients show great potential in the field of personalized sports nutrition. Research based on genomics and metabolomics is expected to provide customized supplementation strategies for different types of athletes (112, 113), thereby optimizing training outcomes and competitive performance. By elucidating the molecular mechanisms of natural ingredients, sports nutrition science can offer scientifically grounded personalized nutritional interventions that both enhance athletic performance and accelerate the recovery process.

#### 4.2.2 Bioactive food components and their metabolic regulation

The biological mechanisms by which food components regulate metabolism are receiving increasing attention, and their application value in sports nutritional supplements is particularly prominent, especially in optimizing athletic performance, promoting recovery, and maintaining health. Many natural food components possess bioactive properties and can regulate energy metabolism, lipid metabolism, and glucose metabolism through multiple pathways, significantly impacting athlete performance.

As shown in Table 5, polyphenolic compounds (such as catechins in green tea and proanthocyanidins in grape seeds) not only possess potent antioxidant effects but also improve lipid metabolism and enhance exercise endurance by inhibiting adipocyte proliferation and promoting fatty acid oxidation (114). These components help reduce fat accumulation and increase the rate of fat oxidation, thereby improving energy utilization efficiency during exercise. Additionally, polyphenols enhance insulin sensitivity by modulating insulin signaling pathways, helping to maintain stable blood glucose levels and optimize energy supply during exercise.

**Table 5:** Metabolic regulation mechanisms of functional food ingredients and their physiological benefits.

Food ingredients	Sources	Mechanism of action	Physiological benefits
Polyphenols	Green tea catechins, grape seed proanthocyanidins	Regulates fat metabolism and insulin pathways	Improve lipid metabolism, increase energy efficiency, improve insulin sensitivity
Dietary fibre	Plant foods	Slows down sugar absorption, regulates the balance of intestinal flora	Stabilise blood glucose levels, enhance immune function, reduce chronic inflammation
Phytosterols	Soy sterols, pepper sterols	Competes with intestinal cholesterol for absorption	Reduce total cholesterol and LDL-C, improve cardiovascular health
Omega-3 fatty acids	Fish oil	Educes oxidative stress and regulates metabolic pathways	Reduce oxidative stress, promote protein synthesis
Curcumin	Turmeric	Regulates nNOSμ and FoxO3a pathways	Reduce protein degradation, relieve muscle damage and inflammation

Dietary fiber is also crucial for maintaining exercise performance, particularly in improving gastrointestinal health and stabilizing blood glucose levels. Studies have shown that dietary fiber can delay carbohydrate absorption, maintain stable blood glucose levels during exercise, and prevent sharp fluctuations, thereby enhancing endurance performance (115). Furthermore, dietary fiber supports gut microbiota balance, which can influence overall metabolic health—including enhanced immune function and reduced chronic inflammatory responses (116). These effects are vital for the long-term health of athletes.

Phytosterols (such as soy sterols and pepper sterols) have demonstrated significant effects in improving lipid profiles and reducing cholesterol levels (117). By competing with cholesterol for absorption in the intestines, phytosterols reduce cholesterol absorption and promote its excretion, effectively lowering total cholesterol and low-density lipoprotein cholesterol (LDL-C) levels in the blood. This is particularly important for cardiovascular health, as it not only enhances endurance but also improves circulatory efficiency during exercise, thereby boosting athletic performance.

Additionally, certain natural food components (such as omega-3 fatty acids in fish oil and curcumin in turmeric) help alleviate post-exercise muscle damage and inflammation by reducing oxidative stress and inflammatory responses. Omega-3 fatty acids and curcumin act through multiple signaling pathways: they inhibit reactive oxygen species (ROS) produced by NOX-2 and mitochondria, while promoting protein synthesis via the HSP70 and Akt/mTOR/p70S6K pathways. Curcumin also reduces protein degradation by modulating the nNOSμ and FoxO3a pathways. These mechanisms collectively work to prevent and mitigate exercise-induced muscle damage (118).

These ingredients can alleviate muscle inflammation caused by high-intensity training, relieve post-exercise muscle soreness, and reduce fatigue during the recovery period. This accelerates the recovery process, enabling athletes to perform subsequent training more efficiently.

In summary, incorporating bioactive food ingredients into sports nutrition provides a multi-dimensional solution for enhancing athletic performance, accelerating recovery, and promoting overall health. Through their diverse biological mechanisms, these natural components precisely meet the physiological needs of athletes, paving the way for optimizing training outcomes and competitive performance.

## 5 From laboratory to sports practice applications

### 5.1 Efficacy of sports nutritional supplements for team sport athletes

Team sports (such as soccer, basketball, rugby) typically feature high-intensity intermittent exercise, requiring athletes to repeatedly sprint, change direction, jump, and take short breaks during competition. This exercise pattern places extremely high demands on the athletes’ phosphagen system, glycolytic system, and aerobic oxidation system, specifically manifesting as an urgent need for repeated sprint ability, stable technical decision-making, and rapid post-match recovery (119). Therefore, the application of sports nutritional supplements in team sports must precisely target these needs, and their efficacy has been widely studied and validated in practice. Although sports nutrition supplements are widely practiced, the evidence for the effectiveness of many specific ingredients in professional players is still mostly from small samples or heterogeneous studies, and their generalisability needs to be combined with individualised assessment.

#### 5.1.1 Soccer players with high demands for endurance enhancement and accelerated recovery

**Figure 6:**
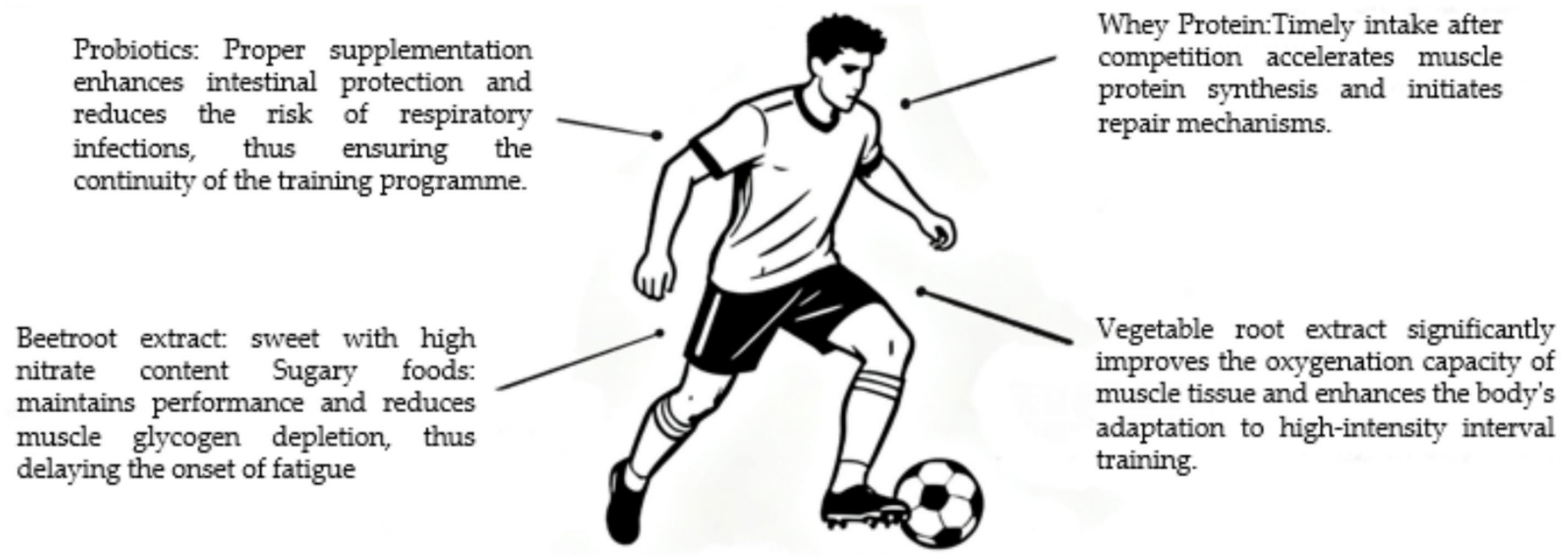
Summary of key nutritional supplement strategies and their proposed benefits for soccer players. The chart categorizes supplements based on their primary intended use (e.g., endurance, recovery, immunity) within the context of soccer’s physical demands.

Soccer matches typically last around 90 min, with players covering an average distance of 10–12 kilometers, including frequent high-intensity activities like rapid sprints. The focus of their nutritional strategy lies in maintaining stable blood glucose levels, delaying central nervous system fatigue, and promoting post-match physical recovery. Pre-match and in-match intake of carbohydrate-containing foods (such as sports drinks, energy gels) has become a standard supplementation practice for professional players (120). This mechanism works by providing an external energy source to sustain performance, reducing muscle glycogen depletion and thereby delaying the onset of fatigue. Research confirms that carbohydrate intake can significantly improve soccer players’ explosive power and movement range during the latter stages of a match. Furthermore, beetroot extract, rich in high concentrations of nitrate, has now become a widely adopted nutritional supplement among professional players (121). This mechanism relies on the nitrate-nitrite-NO pathway to enhance muscle oxygenation and efficiency. This allows athletes to reduce oxygen consumption under the same exercise load, thereby enhancing their physiological adaptation to high-intensity interval training (122). This physiological effect is crucial for soccer players to maintain sprinting and defensive performance in the final stages of a match. To address the issues of muscle fiber microdamage and impaired immune function faced by soccer players during congested fixture schedules, timely post-match intake of whey protein is recommended to accelerate muscle protein synthesis and initiate repair mechanisms (123). Additionally, appropriate probiotic supplementation can strengthen gut barrier function, effectively reducing the risk of respiratory tract infections, thereby ensuring the continuity of training programs (see Figure 6).

#### 5.1.2 Optimizing for basketball players with high demands for explosive power and agility

**Figure 7:**
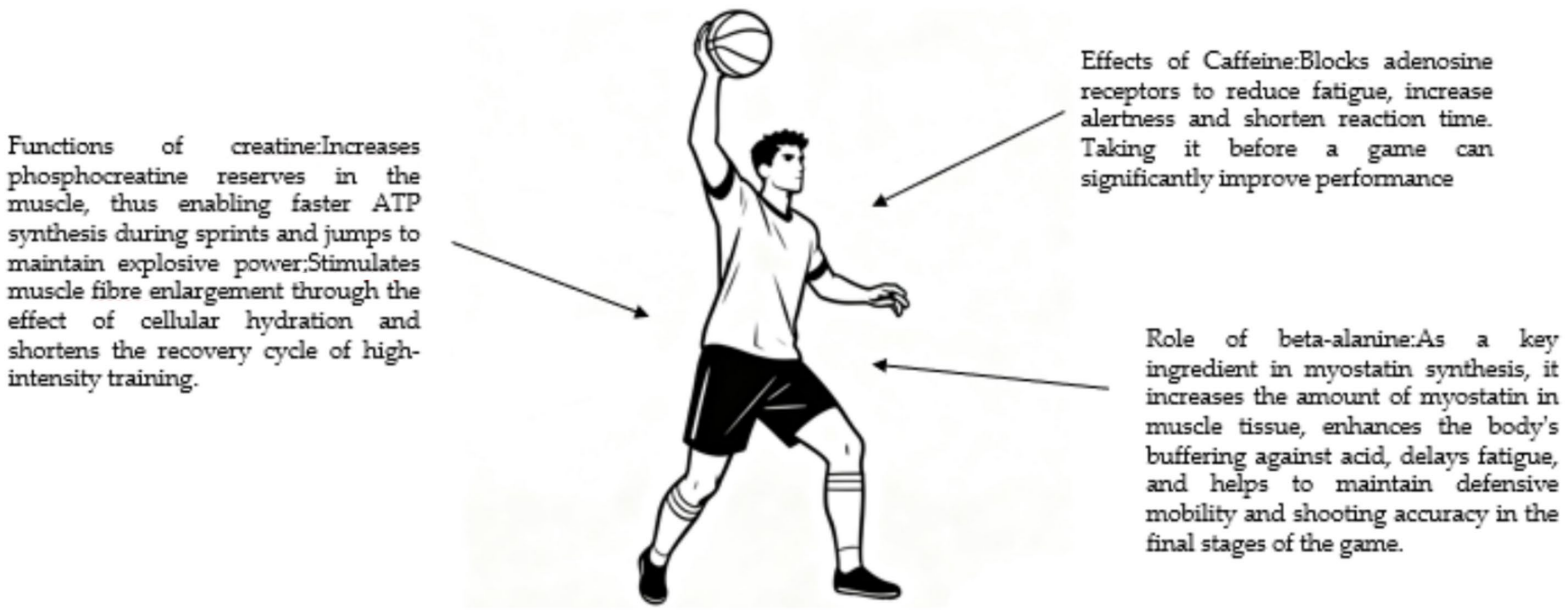
Overview of sports nutrition supplements commonly utilized to address the physiological demands of basketball. Supplements are linked to specific performance or recovery goals relevant to the sport’s intermittent high-intensity nature.

Basketball involves numerous short-distance sprints, jumps, and intermittent pauses, placing high demands on the phosphagen energy system, muscle buffering capacity, and neuromuscular coordination. As a classic nutritional supplement for enhancing explosive power, creatine plays a critical role in short-term, high-intensity exercise. Creatine supplementation, by increasing intramuscular phosphocreatine stores, is critical for maintaining explosive power throughout a basketball game (124). Additionally, creatine may stimulate muscle fiber growth through cellular hydration effects and shorten recovery periods after intense training. During the high-intensity confrontations in basketball, anaerobic metabolism produces excess hydrogen ions, leading to decreased muscle pH and exercise-induced fatigue. Supplementation with *β*-alanine (as discussed in Section 2.1.2) can enhance intramuscular buffering capacity, helping athletes maintain performance in the latter stages of a game (125). This mechanism helps athletes maintain defensive movement speed and shooting accuracy in the later stages of a game. Moderate caffeine intake can block adenosine receptors, alleviating fatigue, enhancing alertness, and shortening reaction times (126). Basketball requires athletes to maintain high levels of concentration and make quick decisions, and pre-game consumption of caffeine-containing substances can significantly improve their competitive state (see Figure 7).

#### 5.1.3 Supporting rugby players with high demands for strength and collision recovery

**Figure 8:**
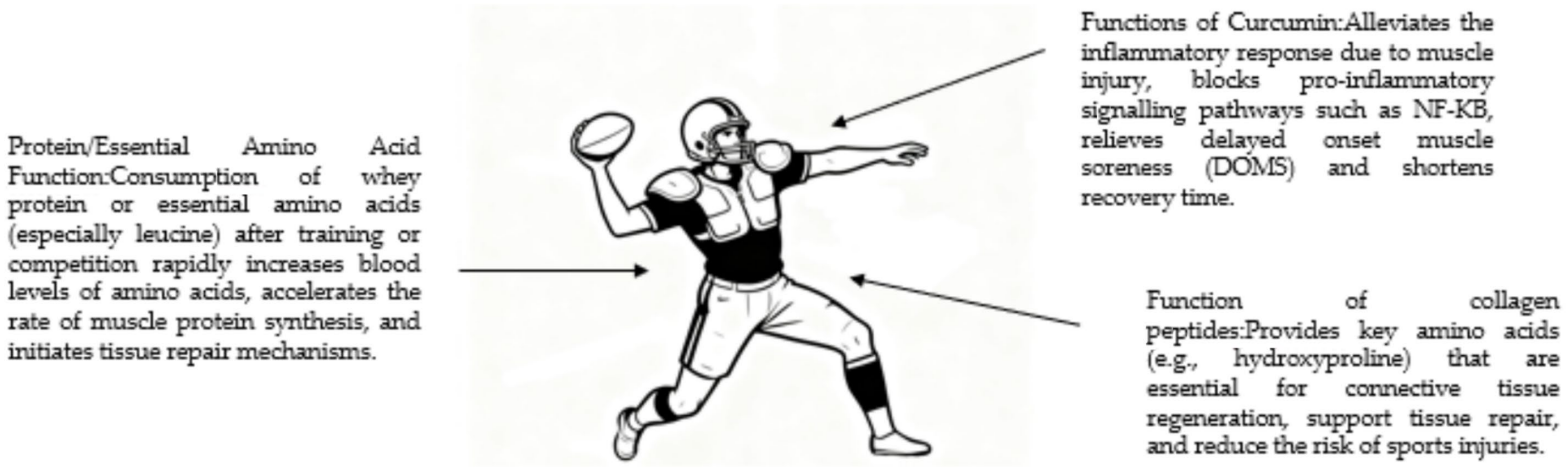
Nutritional supplement strategies aligned with the physical challenges of rugby. The figure maps specific supplements to goals such as supporting muscle repair after collision, managing inflammation, and promoting joint health, reflecting the sport’s unique strength and contact demands.

Rugby is known for its intense physical confrontations, placing high demands on athletes’ strength, instantaneous explosive power, and muscle recovery capabilities after injury. Frequent physical collisions during training and matches can cause significant microtrauma to muscle fibers and accelerate protein catabolism, making supplementation with protein and essential amino acids particularly important (127). The dietary focus for rugby players is to promote muscle protein synthesis and inhibit its degradation. Consuming whey protein or essential amino acids (especially leucine) immediately after training or competition can rapidly increase blood amino acid levels, significantly accelerate muscle protein synthesis, and initiate tissue repair mechanisms (128). Additionally, muscle damage caused by rugby is often accompanied by significant inflammatory responses, which can be alleviated by curcumin (129). As a natural compound with notable anti-inflammatory and antioxidant properties, curcumin effectively promotes bodily repair, primarily by blocking pro-inflammatory signaling pathways such as NF-κB, thereby alleviating exercise-induced delayed onset muscle soreness, significantly shortening recovery time, and helping athletes return to high-intensity training more quickly (130). The career longevity of rugby players is closely linked to joint health. During intense exercise, ligaments, tendons, and cartilage endure substantial loads. Recent studies suggest that collagen peptide intake may supply key amino acids (such as hydroxyproline) required for connective tissue regeneration, aiding tissue repair and reducing the likelihood of sports injuries (see Figure 8).

### 5.2 Efficacy of sports nutritional supplements for individual event athletes

Compared to team sports, the characteristics of individual events are more singular and extreme, providing an ideal scenario for implementing highly personalized nutritional supplementation strategies. The nutritional needs of individual event athletes are determined by the physiological and biochemical basis of their sport, and the efficacy of supplements is reflected in the precise regulation of specific metabolic pathways. This section uses marathon running, weightlifting, and gymnastics as examples to elaborate in detail on how sports nutritional supplements exert their personalized effects tailored to the demands of different individual events.

#### 5.2.1 Maximizing endurance and delaying limits for marathon runners with high demands

**Figure 9:**
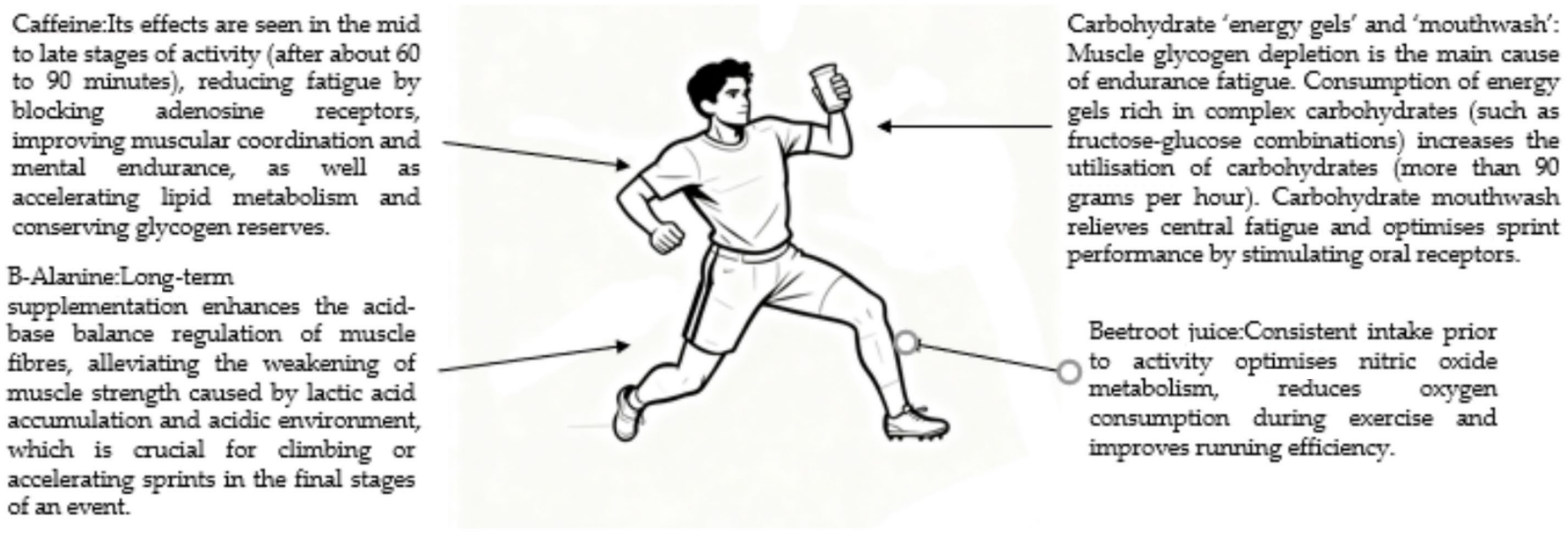
Framework for nutritional support in marathon running, highlighting supplementation timing and goals. The timeline illustrates the application of different supplements before, during, and after a race to address energy depletion, central fatigue, and metabolic efficiency specific to prolonged endurance exercise.

As a typical prolonged endurance event, the physiological mechanism of marathon running primarily depends on aerobic metabolic efficiency, energy substrate allocation, and electrolyte homeostasis (131). Its nutritional intervention aims to delay the onset of fatigue, ensuring that athletes can maintain a continuous energy supply throughout the several-hour race. Among these interventions, carbohydrate “energy gels” and “mouth rinses” are particularly critical, as muscle glycogen depletion is the main cause of endurance fatigue (132). During competition, regular intake of energy gels rich in complex carbohydrates (such as fructose-glucose combinations) can significantly enhance carbohydrate utilization (exceeding 90 grams per hour) by leveraging different absorption pathways in the digestive tract, thereby continuously supplying the energy required for exercise (133). Concurrently, the innovative method of carbohydrate mouth rinsing works by stimulating oral receptors to activate specific brain regions, temporarily alleviating central fatigue. This approach avoids placing stress on the digestive system while optimizing competitive performance during sprint phases. As a neurostimulant, caffeine becomes effective in the middle to later stages of a marathon (approximately 60–90 min after the start) by blocking adenosine receptors to reduce the perception of fatigue, while simultaneously enhancing muscle coordination and psychological endurance (134). This substance also functions to accelerate lipid metabolism, aiding in the conservation of the body’s glycogen reserves. Research indicates that sustained pre-race intake of nitrate-rich beetroot juice can optimize nitric oxide metabolism, thereby reducing exercise oxygen consumption and improving running efficiency. Meanwhile, long-term supplementation with *β*-alanine can strengthen the acid–base regulatory function of muscle fibers, effectively mitigating the decline in muscle strength caused by lactate accumulation and acidic environments, which is particularly crucial during climbing or acceleration phases in the latter part of the race (see Figure 9).

#### 5.2.2 Weightlifters with high demands for breaking strength limits and promoting synthesis

**Figure 10:**
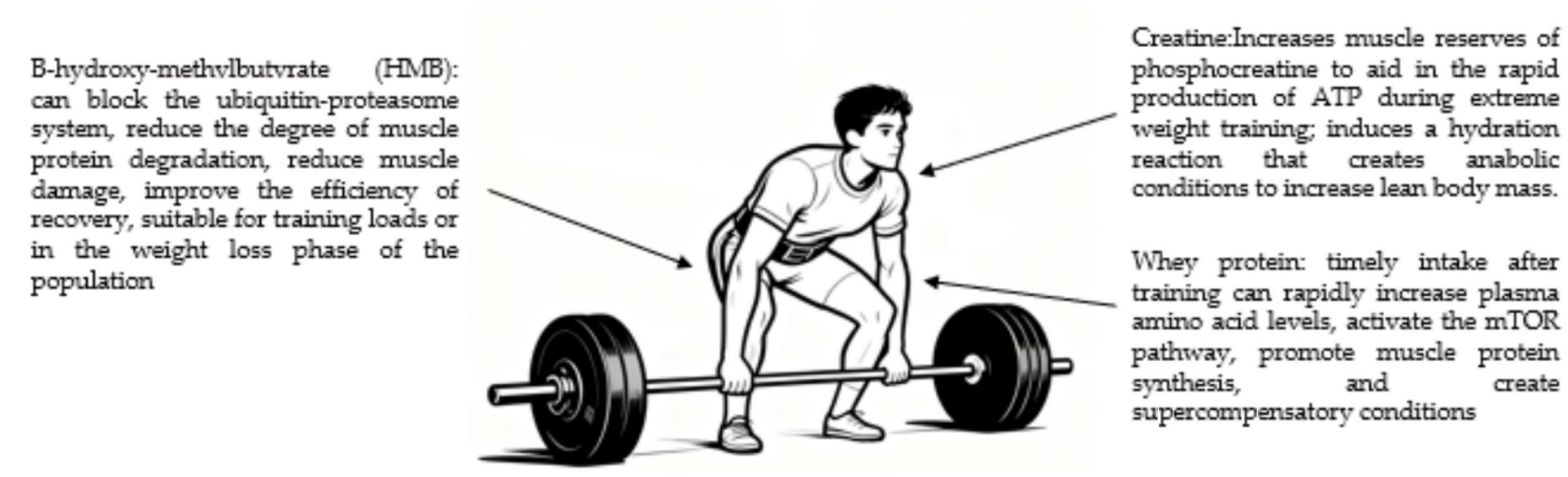
Key supplements and their primary roles in supporting weightlifting training and adaptation. The focus is on nutrients that enhance phosphagen system function, stimulate muscle protein synthesis, and reduce protein catabolism to support strength and power development.

In weightlifting, athletes need to release maximum strength in an instant, which primarily relies on the phosphagen energy system. The key objectives of post-competition nutritional interventions include accelerating muscle tissue repair and optimizing protein synthesis efficiency (135). As a benchmark supplement for strength training, creatine supports the phosphagen system, enabling rapid ATP regeneration during maximal lifts (136). Creatine intake by weightlifters can significantly increase the storage of phosphocreatine in muscles, which helps rapidly generate ATP during maximum weight training. Meanwhile, the hydration effect induced by creatine creates more favorable anabolic conditions, synergizing with strength training to continuously increase lean body mass (137). High-intensity strength training causes micro-damage to muscle fibers, at which point the body urgently requires protein raw materials for repair. Timely intake of whey protein after training can quickly elevate plasma amino acid levels, significantly activate the mTOR pathway, and thereby greatly enhance muscle protein synthesis efficiency, creating conditions for subsequent supercompensation. As a metabolite of leucine, *β*-hydroxy-β-methylbutyrate (HMB) primarily exerts anti-catabolic effects. This substance can block the ubiquitin-proteasome system, effectively reducing muscle protein degradation during high-volume training, alleviating muscle tissue damage, and significantly improving recovery efficiency. This characteristic makes it particularly suitable for athletes with heavy training loads or those in weight reduction phases (see Figure 10).

#### 5.2.3 Gymnasts with high demands for ensuring skill execution and precise weight control

**Figure 11:**
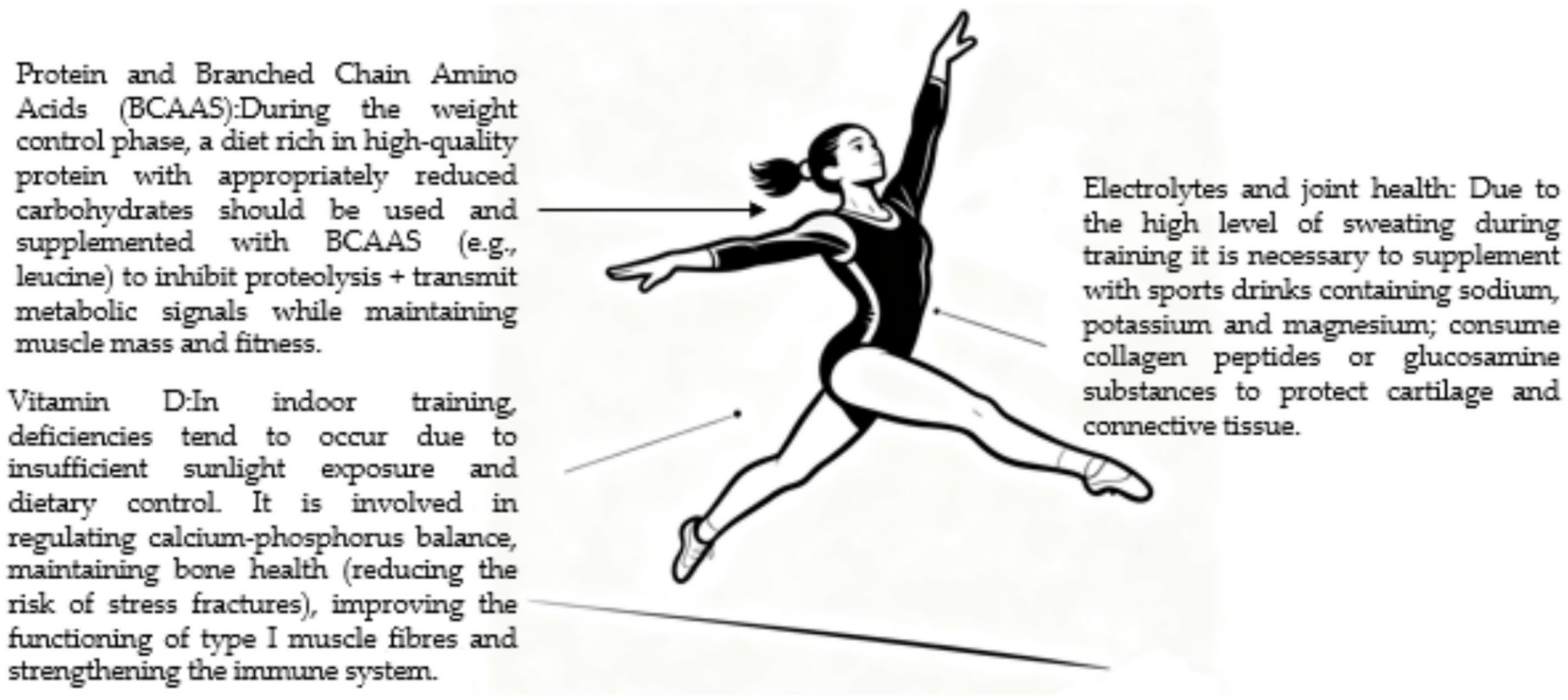
Nutritional priorities and supplement considerations for gymnasts, balancing performance and physique goals. The diagram highlights strategies for maintaining electrolyte balance, supporting joint health, ensuring micronutrient sufficiency (e.g., vitamin D), and preserving muscle mass during energy-restricted phases.

Sports such as gymnastics and diving impose strict standards on explosive power, neuromuscular coordination, and flexibility, while requiring precise control of body fat percentage and weight. Their dietary plans must maintain relatively low body weight while ensuring training effectiveness, accelerating physical recovery, and reducing sports injuries (138). Due to long training durations and significant sweating, even minor fluid loss or electrolyte imbalance can impair concentration, movement coordination, and spatial perception, thus emphasizing the importance of electrolyte balance and joint maintenance (139). Given that high-intensity exercise leads to electrolyte loss, timely supplementation with sports drinks containing minerals such as sodium, potassium, and magnesium is essential. Meanwhile, continuous intense impacts place heavy burdens on joints, and intake of collagen peptides or glucosamine substances may positively affect the maintenance of cartilage and connective tissues, aligning with the needs of gymnasts for preventing chronic injuries (140). Gymnasts, who train indoors for extended periods with insufficient sun exposure combined with strict dietary control, are highly susceptible to vitamin D deficiency. This nutrient not only participates in regulating calcium-phosphorus balance and maintaining bone health (reducing the risk of stress fractures) but also improves type II muscle fiber function and enhances the immune system. Maintaining appropriate vitamin D levels plays a crucial role in enhancing specialized training outcomes. During weight control phases, insufficient energy intake can easily lead to muscle tissue loss (141). Adopting a diet rich in high-quality protein with appropriately reduced carbohydrates, supplemented with branched-chain amino acids (BCAAs), becomes particularly important. Among these, branched-chain amino acids such as leucine can be directly absorbed and utilized by muscle tissue, serving dual functions under energy-deficient conditions: both inhibiting protein breakdown and transmitting metabolic signals, enabling athletes to effectively maintain muscle mass and physical performance during fat loss processes (see Figure 11).

## 6 Current challenges and future prospects of sports nutritional supplements in athletics

### 6.1 Current challenges of sports nutritional supplements in athletics

Although innovations in food science have brought unprecedented opportunities for the development of sports nutritional supplements, the field still faces numerous serious and urgent scientific and regulatory challenges from laboratory research to widespread application in sports practice (142). Clearly understanding these challenges is a necessary prerequisite for promoting the standardization, precision, and safety of the field (143).

#### 6.1.1 Gaps in scientific validity and evidence base

Most sports nutrition products currently on the market claim benefits that often lack sufficient scientific evidence. Products labeled with “proprietary blends,” in particular, frequently obscure the exact amounts of their ingredients, a practice especially common in pre-workout supplements and multi-ingredient formulations. Due to the lack of transparent product information, researchers, coaches, and athletes struggle to verify the efficacy of these products or confirm whether active ingredients—such as beta-alanine, which requires daily supplementation of several grams to elevate muscle carnosine levels—are present in adequate amounts or if ineffective substances have been added. This lack of transparency severely hampers evidence-based decision-making. Existing research is notably limited and difficult to generalize, as most studies on nutritional supplements are conducted under laboratory conditions, involving short-term observations of untrained individuals or animal models (144). Given the unique demands of long-term, high-intensity training among elite athletes, the applicability of current research findings remains questionable. For instance, ingredients that improve insulin sensitivity in sedentary populations may not necessarily enhance running performance in marathoners. Due to significant variations in subjects, exercise types, dosages, and durations, many laboratory findings are challenging to translate effectively into real-world sports settings (145). Current mechanistic research also has clear limitations. Although Chapter 4 highlights the value of methods like metabolomics in elucidating mechanisms, the precise molecular pathways and metabolic mechanisms of many traditional and emerging supplements remain inadequately understood. For example, certain plant extracts, despite their promising “multi-target” properties, complicate scientific validation, making it difficult to accurately identify core active components and primary mechanisms of action.

#### 6.1.2 Safety, purity, and regulatory gaps

Athletes’ health and career development face serious challenges, with safety risks from sports nutrition products being particularly significant. Since these products are generally classified as dietary supplements rather than drugs in most countries, they are exempt from rigorous pre-market safety and efficacy reviews, leading to frequent issues of adulteration and contamination. Inadequate regulation has resulted in notable quality concerns, the most severe being the inclusion of banned substances (146). In some bodybuilding or fitness products, testing has revealed the presence of prohibited compounds such as anabolic steroids and methylsynephrine. Professional athletes who unknowingly consume contaminated products are at high risk of testing positive for banned substances, which can have devastating consequences for their careers (147). Even when products are not contaminated, there may be significant discrepancies between labeled and actual ingredient amounts, ranging from under-dosing to excessive levels. Marketing claims like “instant results” or “breakthrough technology,” commonly seen in the market, are often unsupported by rigorous clinical trials. Such claims not only mislead consumers but also reflect a serious disregard for the safety of dietary supplements (148).

#### 6.1.3 Individual differences and response heterogeneity

Current practices in sports nutrition suffer from an overreliance on standardized supplementation protocols, failing to adequately account for significant individual differences in athletes’ physiological and biochemical markers. Research indicates that genetic polymorphisms (such as those affecting caffeine metabolism and nitric oxide production) and gut microbiota characteristics collectively determine variations in athletes’ responses to nutritional supplements. The efficacy of probiotics, in particular, largely depends on the user’s pre-existing gut microecological environment (149). While personalized nutrition represents the future direction, its practical application still faces numerous challenges. Due to significant individual differences among athletes, predicting the effects of specific nutritional supplements is highly challenging (150). Studies have shown that in supplementation trials involving beetroot juice (containing nitrates), creatine, and caffeine, a considerable proportion of users exhibit no significant benefits. Blindly adopting team-based or popular supplementation protocols without personalized assessment not only leads to resource waste but may also prevent many athletes from achieving expected results, thereby affecting their training motivation (151).

#### 6.1.4 Conflict between commercialization and scientific integrity

In the field of sports nutrition, economic interests often conflict with scientific integrity. Due to publication bias, studies confirming product effectiveness are more likely to be published and disseminated than those with negative conclusions, leading to potential exaggeration of certain products’ actual efficacy in both scientific literature and public perception. Meanwhile, corporate-sponsored research projects may exert potential influence on experimental design and result analysis, thereby raising concerns about conflicts of interest. During information dissemination in sports, coaches and athletes face diverse information sources—such as social media platforms, commercial promotions, and peer recommendations—where content typically lacks professional oversight and contains substantial misinformation and misleading guidance (152). Consequently, rigorously peer-reviewed scientific findings struggle to reach target audiences accurately. As previously mentioned, the phenomenon where “athletes’ excessive reliance on nutritional supplements may lead to prohibited substance use” fully demonstrates the negative impact of cognitive biases (153).

#### 6.1.5 Scientific and regulatory barriers in technological optimization

The translation of food science and technology faces multiple challenges in scientific validation and technological optimization, primarily stemming from the complexity of food ingredients, variability in experimental conditions, difficulties in process optimization, and the need for transparency in scientific validation. First, the complexity of food ingredients makes it difficult to replicate laboratory research results in actual production—factors such as raw material sources, processing techniques, and storage conditions can all affect the bioactivity and functional properties of food (154). Therefore, more precise experimental models are needed to simulate different production and storage conditions to achieve reliable assessments. Second, optimizing processing parameters such as temperature, time, and pressure requires a balance between food safety, nutritional value, and sensory characteristics, which demands a deep understanding of the physical, chemical, and biological properties of food (155). For example, heat treatment may lead to the degradation of nutrients or active ingredients, while different processing methods can alter the structure and texture of food (156). The core challenge lies in enhancing nutritional value while ensuring quality and safety. Additionally, as public concern for food safety and nutrition increases, the transparency and reproducibility of scientific validation have become increasingly important. To ensure the reliability of research results, scientists must eliminate potential biases and misleading outcomes through rigorous experimental design and data analysis, thereby enhancing research credibility and building consumer trust (157).

### 6.2 Future directions of sports nutritional supplements in athletics

To address current challenges in scientific validity, individual responses, and safety regulation, the future development of sports nutritional supplements will increasingly rely on the integration of interdisciplinary technologies and the innovation of concepts. Future directions will focus on achieving precision in nutritional interventions through cutting-edge technologies, enhancing the effectiveness of programs through comprehensive digital management, and ensuring the green development of the industry by developing sustainable raw materials. These directions must also contend with and seek to resolve the significant safety and translational challenges associated with promising but complex technologies like nanotechnology.

#### 6.2.1 Deep integration of artificial intelligence and digital health technology

As a core technological engine, artificial intelligence and digital healthcare will transcend their traditional auxiliary roles, bringing breakthrough progress in overcoming issues such as insufficient clinical evidence and individual efficacy differences. Through AI technologies like natural language processing, vast amounts of scientific literature, clinical trial data, and sports medicine archives can be intelligently analyzed and integrated, constructing a real-time updated network of scientific evidence, fundamentally changing the previously unclear efficacy of “compound supplements.” Based on machine learning algorithms, systems can comprehensively analyze categories of nutritional supplements, athletes’ physical data, training intensity, and external conditions to dynamically generate personalized supplementation plans, adjusting the type, dosage, and timing of supplements in real time. Through smart wearable devices (including dynamic glucose monitors, fluid analysis patches, and bioelectric sensors) that collect real-time physiological indicators (such as changes in glucose metabolism, mineral depletion, and muscle fatigue), combined with AI analysis technology, the nutritional status of athletes can be tracked in real time with alerts for abnormalities. This technology upgrades nutritional interventions from pre-set static plans to dynamic regulation based on real-time physiological data feedback. For example, by monitoring changes in sodium ion concentration in sweat, intelligent systems can dynamically optimize athletes’ electrolyte replenishment strategies. Currently, the application of AI in the field of sports nutrition personalisation is mostly at the stage of algorithm development and pilot studies. For example, studies have attempted to use machine learning to predict athletes’ responses to carbohydrate requirements. However, there is a gap in large-scale proof-of-concept trials of AI dynamic nutritional regulation systems integrating multi-dimensional real-time data in elite sports.

#### 6.2.2 Personalized nutrition based on multi-omics profiling

Personalized nutrition programs will evolve beyond the current simplistic models that only consider body composition and exercise type, advancing into a refined stage supported by multi-omics technologies—including genomics, metabolomics, and microbiome analysis. This represents an extension of the principles discussed in Chapter 4. Shifting from a “one-size-fits-all” approach to “tailor-made” solutions, the construction of an athlete’s unique multi-omics profile enables accurate prediction of their individual responses to various nutritional supplements. For example, genetic testing can distinguish whether an individual metabolizes caffeine rapidly or slowly; gut microbiome analysis can assess their ability to convert dietary nitrate into nitrite; and baseline metabolomic data can predict the extent to which creatine supplementation enhances muscle anabolism. By integrating metabolomics with artificial intelligence, researchers can identify “non-responder” phenomena in advance, avoiding unnecessary supplementation. This approach selects key dynamic biomarkers closely linked to athletic performance, fatigue levels, and recovery status based on physiological cycle characteristics. By dividing an athlete’s training year into more precise “nutritional microcycles,” coaches and nutrition experts can flexibly adjust nutritional strategies in real time according to metabolic indicators—such as inflammation, oxidative stress, and muscle damage—thereby optimizing recovery outcomes. Initial exploratory studies have been conducted on personalised nutrition programmes based on multi-omics, such as genomics to predict individual differences in response to caffeine or nitrates. However, there is no proven framework for the implementation of “whole-ome” personalised programmes that integrate genomic, metabolomic and microbiomic data to guide the long-term nutritional cycle of elite athletes, and there is a lack of randomised controlled trials that demonstrate significant superiority over traditional approaches.

#### 6.2.3 Innovative ingredients from sustainable and precision fermentation sources

To address ecological conservation pressures and consumer demand for natural formulations, food technology is driving transformations in the raw materials sector. The future supply of protein in sports nutrition products will exhibit a trend toward diversification. In addition to traditional animal proteins, novel protein sources such as insect-extracted protein, algae-derived protein, and whey and casein produced through precision fermentation technology will play a significant role. These materials not only offer better ecological friendliness but can also optimize amino acid composition through genetic modification techniques, thereby surpassing conventional proteins in functionality. For plant-based active substances with low extraction rates, such as curcumin, synthetic biology and precision fermentation technologies have opened new pathways. Researchers implant key gene sequences for synthesizing target compounds into microbial carriers (e.g., yeast), utilizing fermentation systems to achieve efficient, controllable, and scalable production. This approach not only overcomes the limitations of traditional agriculture, such as arable land, weather conditions, and chemical agents, but also ensures product purity and batch-to-batch consistency, providing a sustainable solution for the green manufacturing of functional ingredients. Although precision fermentation to produce whey or casein proteins has been realised at laboratory scale and there are start-up companies in product development, there is still limited data from publicly available, peer-reviewed clinical studies on their large-scale application in sports nutrition, sensory acceptance and efficacy in promoting muscle synthesis in comparison to traditional proteins. Research on novel protein sources such as insect proteins in athlete populations is even more lacking.

#### 6.2.4 Regulatory technology (RegTech) and end-to-end traceability

To address safety and trust concerns, the sports nutrition supply chain will integrate regulatory technologies such as blockchain and the Internet of Things. By establishing a credible and transparent system, information from all stages—from raw material cultivation and bio-fermentation to finished product processing and end sales—will be recorded in real time on the blockchain, creating a tamper-resistant, publicly accessible digital record. Coaches and athletes need only scan a product’s QR code to access comprehensive production and distribution records, including purity tests for ingredients and banned substance inspection certificates issued by authoritative institutions. This approach can effectively curb counterfeit and substandard products, rebuilding consumer trust. Leveraging artificial intelligence, regulatory authorities can conduct big data analysis based on user reviews, side effect feedback, and market sampling results collected through the platform, shifting from reactive responses to proactive prevention and enabling real-time monitoring and precise control of product risks.

## 7 Conclusion

The development of sports nutritional supplements has evolved from simple nutrient replenishment into an innovative field deeply integrating food science, exercise physiology, and molecular biology. This review elucidates the full-chain research pathway—from fundamental physiological principles to laboratory innovations (such as microencapsulation and nanotechnology enhancing bioavailability), to biological mechanism validation (e.g., using metabolomics to clarify the action pathways of natural ingredients), and ultimately to precise application in sports scenarios. Research indicates that innovations in food science are the core drivers advancing sports nutrition products. Not only do they address challenges in stabilizing and delivering active ingredients through advanced processing technologies, but they also provide solid scientific foundations for enhancing athletic performance and accelerating recovery by exploring the multiple physiological functions of natural ingredients like plant extracts and probiotics. Concurrently, studies reveal that the efficacy of supplements highly depends on the specific demands of sports disciplines (such as repeated sprint ability in team sports versus extreme endurance or strength in individual events) and individual physiological differences among athletes. However, the field still faces significant challenges. These include inconsistent scientific evidence, heterogeneous individual responses, and incomplete regulatory systems that are ill-equipped for novel applications like nanotechnology—a domain where safety concerns, bioaccumulation risks, and a lack of human trials present substantial translational hurdles. Looking ahead, sports nutrition science is poised to move toward precision and intelligence. AI-driven data mining and personalized recommendations, multi-omics-guided individualized nutrition, sustainable raw materials and synthetic biology enabling source innovation, and blockchain technology ensuring full-chain traceability will collectively build a more efficient, safer, and more personalized new paradigm for sports nutrition. Only through close interdisciplinary collaboration and continuous promotion of scientific translation from the laboratory to the field can the immense potential of food science in sports nutrition be fully unleashed, ultimately empowering every athlete to achieve peak performance and long-term health.
